# Diapause Induction and Termination in *Hyphantria cunea* (Drury) (Lepidoptera: Arctiinae)

**DOI:** 10.1371/journal.pone.0098145

**Published:** 2014-05-30

**Authors:** Chao Chen, XiaoTang Wei, HaiJun Xiao, HaiMin He, QinWen Xia, FangSen Xue

**Affiliations:** 1 Institute of Entomology, Jiangxi Agricultural University, Nanchang, Jiangxi Province, China; 2 Qingdao Entry-Exit Inspection and Quarantine Bureau, Qingdao, Shandong Province, China; University of Texas Southwestern Medical Center, United States of America

## Abstract

The fall webworm, *Hyphantria cunea* (Drury), enters facultative diapause as a pupa in response to short-day conditions during autumn. Photoperiodic response curves showed that the critical day length for diapause induction was 14 h 30 min, 14 h 25 min and 13 h 30 min at 22, 25 and 28°C, respectively. The photoperiodic responses under non-24 h light–dark cycles demonstrated that night length played an essential role in the determination of diapause. Experiments using a short day length interrupted by a 1-h light pulse exhibited two troughs of diapause inhibition and the effect of diapause inhibition was greater in the early scotophase than in the late scotophase. The diapause-inducing short day lengths of 8, 10 and 12 h evoked greater intensities of diapause than did 13 and 14 h. Diapause can be terminated without exposure to chilling, but chilling at 5°C for 90 and 120 d significantly accelerated diapause development, reduced mortality, and synchronized adult emergence. Additionally, the potential for *H. cunea* from the temperate region (Qingdao) to emerge and overwinter under field conditions in subtropical regions (Nanchang) of China was evaluated. Pupae that were transferred to Nanchang in early July showed a 60% survival rate and extremely dispersed pupal period (from 12 to 82 days), suggesting that some pupae may undergo summer diapause. Diapausing temperate region pupae that were moved out-of-doors in Nanchang during October showed approximately 20% overwintering survival; moreover, those pupae that overwintered successfully emerged the next spring during a period when their host plants would be available. The results indicate that this moth has the potential to expand its range into subtropical regions of China.

## Introduction

The fall webworm, *Hyphantria cunea* (Drury) (Lepidoptera: Arctiinae), is native to North America and has invaded many areas of Europe and Asia since 1940 [1]. It was first found in China in 1979 in Dandong city, Liaoning province, and it has successively spread from Liaoning province to the provinces of Shandong, Shanxi, Hebei and Tianjin Municipality [2]. It now has a relatively stable range in the Temperate Region from 32° N to 40° N. There are no records for the species at lower latitudes or under subtropical conditions in China.

The fall webworm is known to feed on over 100 species of deciduous trees in China and causes significant damage to many ornamental trees [3]. *Hyphantria cunea* completes two or three generations per year in China and overwinters on the ground as a diapausing pupa. In recent years, some bivoltine populations have been observed to produce three generations; during these years, September temperatures were especially high [4]. Thus, *H. cunea* is currently considered a major economic pest, and it has the potential for extending its range and causing additional damage.

Diapause is one of the basic means by which insects cope with unfavorable environmental conditions. The diapause processes include diapause induction, maintenance, termination, post-diapause quiescence, and post-diapause development. In most insects, these processes are regulated mainly by abiotic factors [5]–[7], particularly photoperiod and temperature. It is important to understand the influence of photoperiod and temperature on these processes because such information is essential for predicting the initiation and termination of diapause in the field.

Effects of photoperiod and temperature on the induction of diapause [8]–[14] and the physiology of overwintering pupae [15] in *H. cunea* have been studied in detail in Japan. Masaki et al. [8] reported that winter diapause of a population in Yokohama was induced by a short photoperiod during the larval stage. The critical photoperiod for the induction of winter diapause was between 14 h 30 min and 14 h 45 min at 25°C. Gomi and Takeda [12] reported on changes in life-history traits in the fall webworm half a century after its introduction to Japan, and revealed that geographic variation in the critical photoperiod for diapause induction was not a simple cline; the critical photoperiod was longer in populations north of 36° N than in populations south of 36° N. Gomi [14] reported that both the critical photoperiod for diapause induction and the temperature sensitivity of the photoperiodic response were different between the trivoltine populations and their bivoltine counterparts, and the geographic variation in larval and pupal period was positively correlated to the latitude of the original localities of the populations. These results demonstrate that several life-history traits of *H. cunea* adapted to local climatic conditions after the pest’s invasion of Japan.

However, effects of photoperiod and temperature on diapause termination in *H. cunea* have been studied less. Gomi [11] reported that the timing and duration of chilling (5°C) greatly influenced the time required for adult emergence, in which case diapausing pupae chilled for a relatively long period developed to adulthood rapidly. In the present study, we tested the photoperiodic responses of diapause induction and the effects of the diapause-inducing photoperiod and chilling on diapause termination in *H. cunea* under laboratory conditions. In addition, we assessed the potential for pupae from the temperate zone to survive summer conditions and to overwinter in the subtropical region. We did so by transferring temperate region pupae from Qingdao to natural conditions in Nanchang, Jiangxi province during July and again in October, and recording their subsequent patterns of adult emergence. A thorough understanding of diapause induction and termination is still lacking in China, with only one paper reporting diapause characteristics of *H. cunea* [16]. It is important to carry out experiments to predict the seasonal life cycle and the local adaptability of this species in China. Such experiments are also essential for anticipating its ability to invade warmer regions of China.

## Materials and Methods

### Insect Rearing

Because the fall webworm *H. cunea* is an established pest in China, no permits were required for collecting the insect and performing the experiments. All experiments were carried out at the Institute of Entomology, Jiangxi Agricultural University, Nanchang, Jiangxi Province (28°46′ N, 115°49′ E). The voucher specimens (pinned) are placed in the Entomology Museum of Jiangxi Agricultural University.

Approximately 350 full-grown larvae from the first generation of *H. cunea* were collected from their host plant *Platanus acerifolia* in Qingdao city, Shandong province (36° 04′ N, 120° 22′ E). The larvae were reared on *P. acerifolia* in transparent plastic boxes (L×W×H: 17.5×12.5×6.5 cm). Approximately 50 individuals were reared together in each of the plastic boxes and the diet was replenished daily until the larvae reached the pre-pupal state. After the full-grown larvae pupated on the host plant leaves, the pupae were removed from the leaves and transferred to a separate cage (L×W×H: 20×20×30 cm) until the adults emerged. All of the above rearings were carried out under field conditions (outdoors, in a ventilated enclosure surround with gauze screen, so that the temperature, photoperiod and other environmental factors were similar to the field). After the adults emerged, 50 mated pairs were removed and transferred to 5 plastic boxes for oviposition under controlled conditions (25±1°C; LD 15∶9). We added fresh *P. acerifolia* leaves to the boxes when the eggs became black. After hatching, we randomly selected 50–100 larvae from the leaves and transferred them to other plastic boxes with leaves of *P. acerifolia.* All experiments were carried out in climate cabinets (LRH-250-GS, Taihong Medical Instrument Manufacturer, Guangdong, China) equipped with six fluorescent 30-W tubes. The light intensity during photophase was approximately 2.0 W/m^2^.

### Photoperiodic Response

To determine the influence of photoperiod on diapause induction, we reared individuals under 25 experimental conditions: seven day lengths – LD 16∶8, 15∶9, 14∶10, 13∶11, 12∶12, 10∶14 and 8∶10, at each of four temperatures –22, 25, 28 and 31°C (except LD 13∶11 which was only tested at 28°C). Temperatures varied±1°C. We recorded the incidence of pupal diapause under each condition and used these data to determine the critical day length for diapause induction (the day length under which 50% of the individuals entered diapause). Pupae persisting for more than 10 days after the last eclosion of non-diapausing pupae under LD 16∶8 at each temperature were considered to be in diapause. There were at least 30 individuals in each of three replicates for each treatment, but more than 1,000 individuals were reared under LD 12∶12 and 25°C to obtain a sufficient number of diapausing pupae for subsequent experiments on the termination of diapause.

### Photoperiodic Response Experiments under Non-24 H Light–dark Cycles

For most insects, the duration of the dark period of the day-night cycle is a crucial factor in the determination of diapause [17]. Thus, we designed an experiment to explore whether this phenomenon exists in *H. cunea*. In this experiment, larvae were exposed to non-24 h light–dark cycles with constant photophases of 8, 10, 14 and 16 h at 28°C and with scotophases varying from 4 to 24 h. There were at least 30 individuals in each of three replicates for all of the treatments except for the photophase 8 h: scotophase 12 h treatment, which had only one replicate of 32 individuals.

### Night Interruption Experiments in 24 H Photoperiods

To find possible photosensitive phases under 24-h light–dark cycles, the photoperiodic backgrounds of LD 11∶13 (a diapause-inducing photoperiod) and LD 13∶11 (close to the critical day length) were adopted, in which the scotophase was systematically interrupted by a single 1 h light pulse beginning at every successive hour (at 28°C). There were at least 30 individuals in each of three replicates for all of the treatments except for the treatment where the interruption occurred during the sixth hour after dark; this treatment had only one replicate of 37 individuals.

### Effect of Diapause-inducing Photoperiod on Diapause Termination

To examine whether the diapause-inducing photoperiod has a significant influence on the intensity of diapause, 10-day-old diapausing pupae induced by LD 8∶16, LD 10∶14, LD 12∶12, LD 13∶11 and LD 14∶10 at 28°C were transferred to 28°C and LD l6∶8 to terminate diapause. Adult emergence was recorded daily until emergence was completed. The criterion for diapause termination was adult emergence, thus our measurement of diapause duration included the period of post-diapause development.

### Effect of Chilling on Diapause Termination

To determine the effect of chilling on diapause development, 10-day-old pupae that had entered diapause under LD 12∶12 (25°C) were chilled in a refrigerator (constant 5°C±1°C) for 0, 30, 90, 120 and 150 d in continuous darkness (DD). After chilling, the pupae were transferred to 25°C and LD 16∶8 for emergence observation.

### Adult Emergence by the First Generation, Temperate Region Pupae under Subtropical Conditions

Approximately 350 full-grown larvae in the first generation of *H. cunea* were collected on their host plant *P. acerifolia* in late June in Qingdao city (temperate region) and allowed to pupate under field conditions in Nanchang (subtropical region). More than 300 individuals pupated in early July. Then, they were transferred to the Institute of Entomology, Nanchang, Jiangxi Province in early July and were kept under field conditions to determine their pattern of adult emergence. Adult emergence was recorded daily until emergence finished.

### Post-diapause Emergence by the First Generation, Temperate Region Pupae under Subtropical Conditions

To test whether temperate region pupae can survive subtropical winter conditions, approximately 1000 pupae were induced into diapause under LD 12∶12 at 25°C and were moved in early October to the field at the Institute of Entomology, Nanchang, Jiangxi Province to overwinter. We recorded the adult emergence daily the following spring.

### Statistics

Statistical analyses were conducted using SPSS 17.0. Kruskal–Wallis tests followed by Bonferroni post-hoc tests were used to determine whether differences in the duration of diapause among different diapause-inducing photoperiods were significant. Differences in the duration of diapause between different chilling treatments were compared using one-way analysis of variance (ANOVA) followed by multiple comparisons analysis using Tukey’s HSD test. Throughout the text all means are given as±1 SE.

## Results

### Photoperiodic Induction of Pupal Diapause

**Figure 1:**
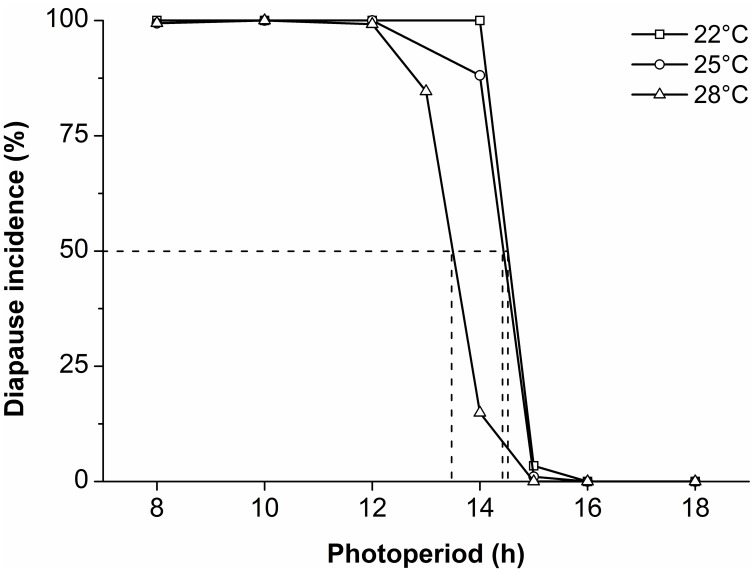
Photoperiodic response curves for the induction of pupal diapause in *H. cunea* at 22, 25 and 28°C (3 replicates, each of at least 30 individuals/treatment; total n = 147–1341).

All of the larvae maintained at 31°C died before reaching the pupal stage. Under the other temperatures, the photoperiodic response curves showed a typical long-day response for the induction of pupal diapause (Fig. 1). More than 80% of individuals entered diapause under the day lengths of 8, 10, 12 and 14 h at both 22 and 25°C, whereas almost all individuals developed without diapause under the day lengths of 15, 16 and 18 h. The incidence of diapause was quite low (18%) under the day length of 14 h at 28°C. In summary, the critical day lengths were 14 h 30 min, 14 h 25 min and 13 h 30 min at 22, 25 and 28°C, respectively.

### Photoperiodic Response Curves under non-24 H Light–dark Cycles

**Figure 2:**
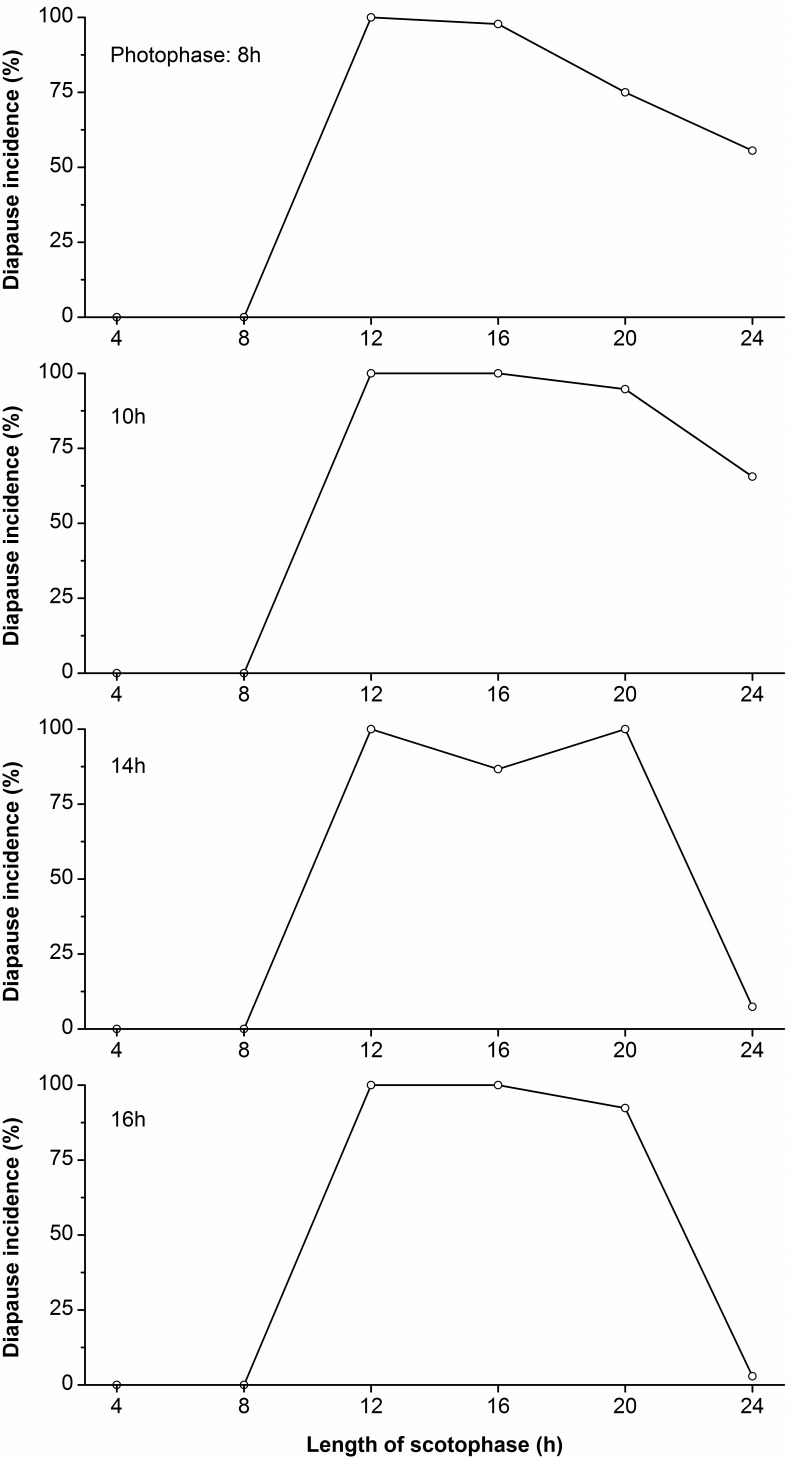
Photoperiodic response curves for the induction of diapause in *H. cunea* under non-24 h light–dark cycles at 28°C. The length of photophase (top left of each panel) was held constant and the scotophase was changed in each experiment (X-axis). (3 replicates, each of at least 30 individuals/treatment; total n = 32–200 for each point).

Regardless of the length of photophase (8, 10, 14 and 16 h), the incidence of diapause was always high when combined with scotophases of 12 to 20 h, suggesting that the dark period is critically important for the determination of diapause (Fig. 2). However, the incidence of diapause decreased markedly when combined with an ultra-long scotophase of 24 h, especially with 14 and 16 h photophases (more than 90% individuals developed without diapause).

### Night Interruption Experiments in 24 H Photoperiods

**Figure 3:**
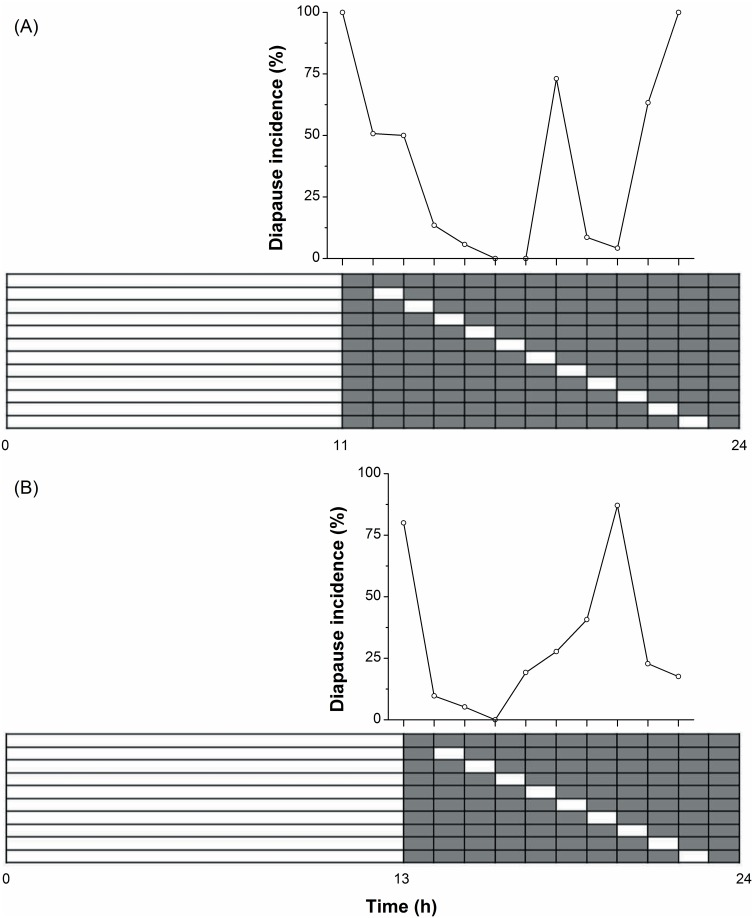
Night interruption for the induction of diapause in *H. cunea* under LD 11∶13 h (A) and LD 13∶11 h (B), 28°C regimes. The scotophase was systematically scanned by 1-h light pulse. (3 replicates, each of at least 30 individuals/treatment; total n = 37–198 for each point).

When the scotophase of the LD 11∶13 treatment (a diapause-inducing photoperiod) was systematically interrupted by a 1-h light pulse, two troughs (the so-called A and B troughs) of diapause inhibition were observed when the light pulses were applied 6–7 h after lights-off and 4–5 h before lights-on, respectively (Fig. 3A), but the first trough (100% development) was slightly deeper than the second trough (96% development). When the scotophase of the LD 13∶11 treatment (close to the critical day length) was systematically interrupted by a 1-h light pulse, the first trough shifted to 4 h after lights-off and the second trough shifted to 2–3 h before lights-on (Fig. 3B). The first trough (100% development) was much deeper than the second trough (83% development). The larvae exposed to 8 h of darkness were relatively insensitive to the light pulse in both treatments, and more than 70% of the individuals entered diapause.

### Effect of Diapause-inducing Photoperiod on Diapause Intensity

**Figure 4:**
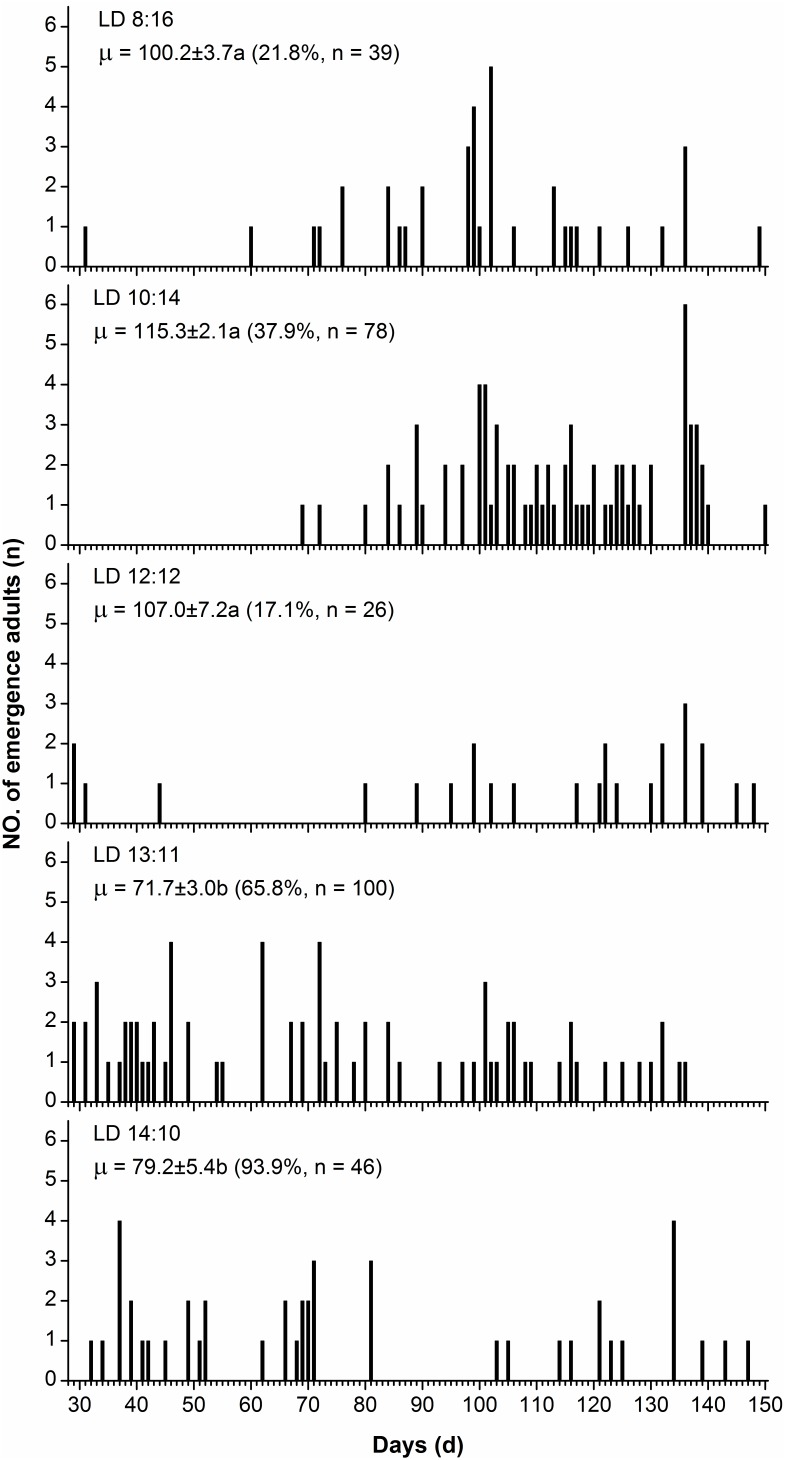
Duration of pupal diapause in *H. cunea* under LD 15∶9 at 28°C, after diapause was induced by different photoperiods at 28°C. Values followed by different letters are significantly different by the Kruskal-Wallis test and Bonferroni multiple comparisons (χ^2^ = 85.29, d.f. = 4, *P* = 0.000<0.01). Values in parentheses are numbers of adults emerged from diapausing pupae and survival rate after diapause.

The duration of diapause was significantly affected by the diapause-inducing photoperiod (Fig. 4). The durations of diapause induced by the short photoperiods of LD 8∶16, LD 10∶14 and LD 12∶12 were significantly longer than those under the relatively long photoperiods of LD 13∶11 and 14∶10 at 28°C (Kruskal–Wallis tests: χ^2^ = 85.29, d.f. = 4, *P* = 0.000<0.01). The mortality of pupae induced into diapause by short day lengths of 8, 10 and 12 h was very high (78.2% for 8 h; 62.1% for 10 h; 82.9% for 12 h), whereas the mortality of diapausing pupae from the day lengths of 13 and 14 h was low (34.2% for 13 h; 6.1% for 14 h).

### Effect of Chilling on Diapause Termination

**Figure 5:**
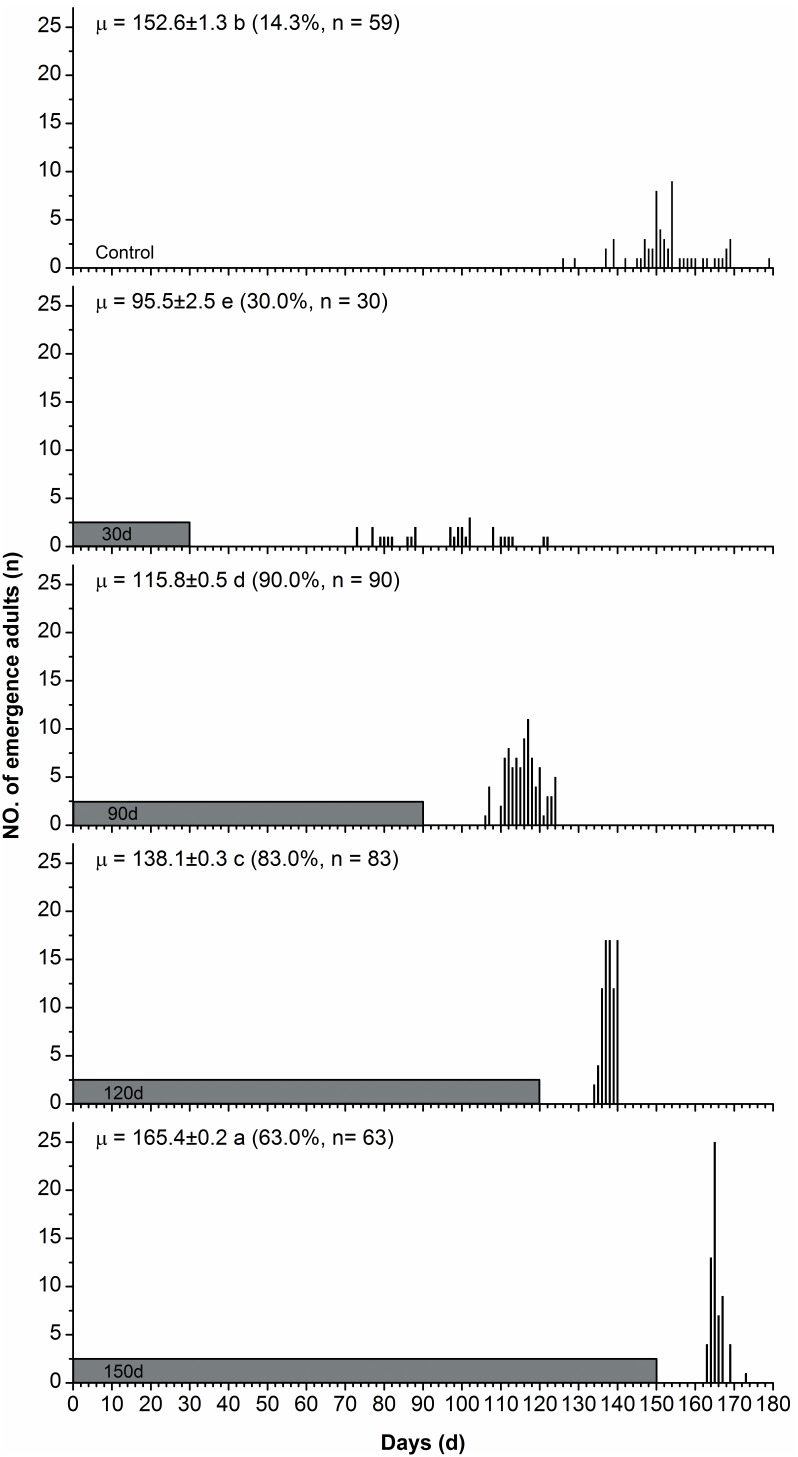
Frequency distribution of adult eclosion in diapausing pupae of *H. cunea*. The diapausing pupae were transferred to LD 15∶9 at 25°C after exposure to 5°C and DD for different numbers of days. The hatched bar indicates the period of cold exposure. Values followed by different letters are significantly different by Tukey’s multiple comparisons (*P*<0.05). Values in parentheses are survival rates after diapause and numbers of adults emerged from diapausing pupae.

Figure 5 shows the frequency distribution of adult eclosion after chilling for different periods. The duration of diapause was significantly influenced by chilling (F _4,320_ = 1851.8, *P* = 0.000<0.01). The duration of diapause in pupae that were not chilled (mean of 152.6 days) was significantly longer than those that were chilled for 30 days (95.5 days), 90 days (115.8 days) and 120 days (138.1 days), showing that chilling significantly accelerates diapause development (*P*<0.05). However, when chilling extended to 150 days, the duration of diapause (165.4 days) was significantly longer than that of the control. It should be mentioned that the mortality of diapausing pupae without chilling was very high, and only 14.3% of the individuals emerged. Similarly, mortality of the diapausing pupae chilled for 30 days was also very high, and only 30% of the individuals emerged. However, those diapausing pupae chilled for 90 and 120 days had higher survival rates (90% and 83%, respectively). The survival rate of diapausing pupae chilled for 150 days was also relatively high (63%). Moreover, chilling played an important role in synchronizing adult emergence. All adults emerged within 18 days for 90 days of chilling, 7 days for 120 days of chilling and 10 days for 150 days of chilling.

### Distribution of Emergence by First Generation, Temperate Region Adults under Subtropical Conditions

**Figure 6:**
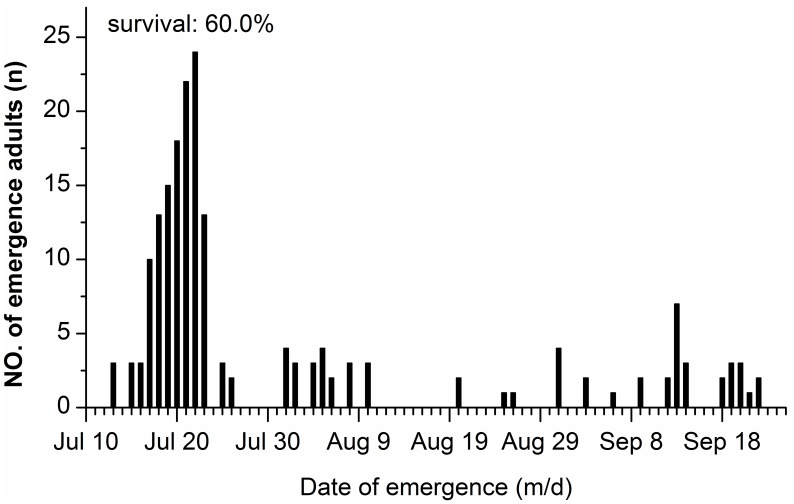
Frequency distribution of adult eclosion in pupae of *H. cunea* under natural conditions in Nanchang. Pupae were collected in Qingdao city and transferred to Nanchang in early July (n = 187).

Eclosion of the first generation adults originally moved as pupae from Qingdao occurred over a very long period under the climatic conditions in Nanchang (Fig. 6). 69.5% of the individuals emerged in mid- to late July with a pupal duration of 12 to 23 days, 11.9% of the individuals emerged in early August with a pupal duration of 30 to 39 days and 19% of the individuals emerged in late August to late September with a pupal duration of 49 to 82 days. In this experiment, the total rate of emergence was 60%.

### Post-diapause Emergence by the First Generation, Temperate Region Pupae under Subtropical Conditions

**Figure 7:**
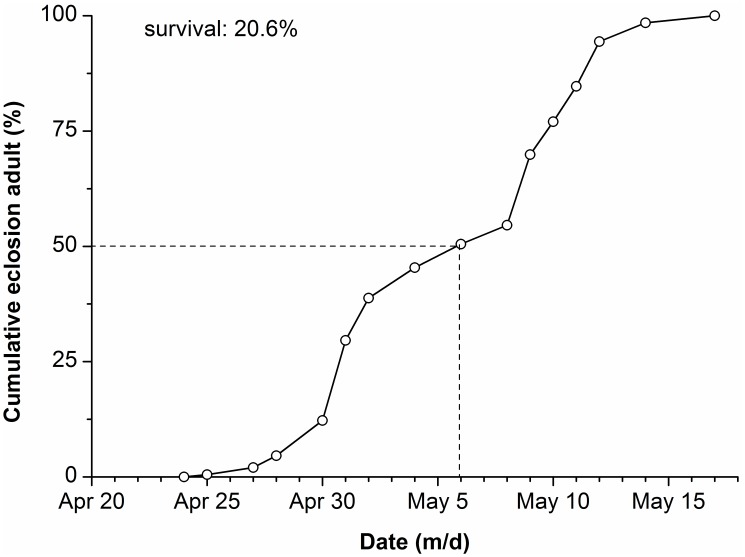
Cumulative rate of diapause termination in naturally overwintering pupae of *H. cunea* in spring of 2012 in Nanchang (n = 196).

Approximately 20.6% of overwintering pupae from Qingdao city terminated diapause and underwent adult eclosion in Nanchang (Fig. 7). Adult emergence began on April 25 with 50% of the individuals emerging by May 6, and the last emergence on May 16.

## Discussion

### Critical Photoperiod for Diapause Induction

Photoperiodic response curves in a population of *H. cunea* from China showed that short day lengths of 8–13 h resulted in more than 80% of individuals entering diapause. This response occurred under a broad range of high temperatures (22–28°C), and the results demonstrate that photoperiod is a crucial factor for timing diapause induction. In our population, the critical day length was 14 h 30 min at 22°C and 14 h 25 min at 25°C. These results are similar to those reported by Masaki [9] for *H. cunea* after its establishment in Japan. His results showed a critical day length of 14 h 35 min at 25°C for the induction of diapause of and that the critical day length was relatively stable over a wide range of temperatures (17 to 25°C.).

The critical photoperiod of our population is equal to the day length recorded on August 18 in Qingdao city (approximately 14 h 30 min including twilight). Thus, we would predict that most of the larvae from the second generation, which hatched under the high temperatures prevailing during August, would result in pupae that entered winter diapause. Additionally, in our study, a number of the larvae that hatched in early August developed without diapause, and produced another (third) generation. According to field observations in Qingdao city, adult emergence of the first generation began in early July, the second generation larvae hatched in mid-July and began to pupate in mid-August and the second generation adults began to emerge in late August. Thus, we conclude that this species is partially trivoltine in Qingdao city.

### Photoperiodic Time Measurement

Almost all of the experiments that have been performed with non-24 h light–dark cycles in insects have shown that the dark period (scotophase) is the decisive phase in the determination of diapause, that is, the photoperiodic clock was only shown to measure the duration of scotophase [18]–[23]. Day length measurement was more important than night length measurement in photoperiodic response for diapause induction only in the linden bug, *Pyrrhocoris apterus* and the zygaenid moth, *Thyrassia penangae* [24], [25]. Like most insects, the photoperiod response for the induction of diapause mainly depended on the length of scotophase in *H. cunea*, because the incidence of diapause was very high in cycles containing a long night (12, 16 and 20 h) regardless of the length of the photophase. However, the incidence of diapause was obviously lower when the long day lengths of 14 and 16 h were combined with a scotophase of 24 h (7.4% and 2.9% diapause) than when the long day lengths of 10 and 12 h were combined with a scotophase of 24 h (55.6% and 65.6% diapause) (Fig. 2). This is an interesting phenomenon, but further experimentation is necessary to determine the significance of the findings.

All of the previously tested cases of photoperiodic response in long-day species were highly sensitive to night interruption, and the long night effect could be reversed by a light break [18], [19]. However, the time at which different species are most sensitive to light pulses varies considerably [26]. In some insects, the time at which they are most highly photosensitive occurs in the early scotophase [23], [27]–[29], the middle of scotophase [22], [30], and the late scotophase [20], [25], [31], [32]. In the cabbage butterfly, *Pieris melete* (an intermediate-day species,), night interruption with a 1-h light pulse at LD 11∶13 (a winter diapause-inducing photoperiod) inhibited the incidence of diapause most effectively in late scotophase, whereas night interruption with a 1-h light pulse placed in early scotophase at LD 12.5∶11.5 (a diapause-inhibiting photoperiod) resulted in the highest intensity of diapause [21]. In the present study with *H. cunea*, night-interruption experiments with a 1-h light pulse at LD 11∶13 and LD 13∶11 exhibited two troughs of diapause inhibition, and the effect of diapause inhibition was greater in the early scotophase than in the late scotophase (Fig. 3). However, when the photoperiodic background of LD 6∶18 was interrupted systematically by a 1.5 h light pulse, only one trough of diapause inhibition was found in *H. cunea*, which occurred at 7.5 h in the darkness [26]. Our results further suggest that the time at which the highest photosensitivity to light pulse occurs may be different even within the same species depending on the experimental conditions.

### Diapause Maintenance and Termination

The diapause-inducing photoperiod has been shown to influence the intensity of diapause in a number of insect species. Nevertheless, the influence of photoperiod during diapause induction on the intensity of diapause could vary between species but also among individuals of the same species [18]. For example, in the fruit flies, *Drosophila auraria* and *D. subauraria*the, the photoperiods with longer scotophases induced more intense diapause than those with shorter scotophases [33], [34]. In the bean bug, *Riptortus clavatus*, a 12 h scotophase evoked a diapause of greater intensity than either longer or shorter scotophases [35]. In the case of *H. cunea*, the diapause-inducing short daylengths of 8, 10 and 12 h induced a more intense diapause than the long day lengths of 13 and 14 h (Fig. 4). Furthermore, the mortality rates of diapausing pupae induced by short day lengths were significantly higher than those of diapausing pupae induced by relatively long day lengths. However, it is difficult to explain this phenomenon. This result may suggest that diapause-inducing short day lengths combined with a high temperature (28°C) have a detrimental influence on diapause development. Further investigation is needed to explain the phenomenon.

It has been shown that chilling is not a prerequisite for the completion of hibernation diapause in many insects, and in some insects diapause completion progresses well at intermediate or high temperatures [36]–[42]. The results in the present study also indicate that diapause can be terminated without exposure to chilling in *H. cunea*. Suggesting that exposure to low temperature is not a prerequisite for the completion of diapause development in this species. Typically, overwintering insects terminate diapause “spontaneously” (without an overt environmental stimulus) during mid to late winter (the cold period). However, they remain dormant and do not undergo post-diapause development (leading to emergence) until after conditions become warm. Thus, the crucial value in the experiment is the amount of time required for emergence after the pupae are transferred to warm conditions. Figure 5 is consistent with results of a typical diapause. It appears that diapause ended in almost all individuals after they had been under cold conditions for 90–120 days. In those that remained under cold conditions longer (150 days), postdiapause development continued to be suppressed and mortality began to rise.

### Field Observations and Risk of Invasion of Warmer Regions in China

One surprising result of this study is the observation of developmental suppression during the summer in a Chinese population of *H. cunea*. The eclosion time from the first generation pupae, which pupated in early July in natural conditions, extended over a very long period from July 13 to September 22 in the climatic conditions of Nanchang (Fig. 6). Of particular interest is that 19% of the pupae emerged from late August to late September with a pupal duration of 49 to 82 days, showing a quiescence or summer diapause. This extremely long pupal period may be an adaptation to the hot summer in Nanchang because the mean daily temperature in July generally exceeded 30°C (with a historical high temperature of 35°C and a historical low temperature of 28°C, according to data in Nanchang) and the daily temperature fluctuated from a low of 24°C to a high of 38°C. A similar developmental suppression in the pupal stage at high temperatures was also found in the black-headed form of *H. cunea* in Missouri, USA, where adult emergence was extended over 60 days when pupae were maintained at 30°C and constant light [43]. However, we do not know whether the observed developmental suppression in the pupal stage in the first generation exists in Qingdao. Therefore, further investigation is needed to understand this scenario. To our best knowledge, this is the first report of the aestivation phenomenon in *H. cunea* in Asia.

The debate about whether *H. cunea* has the ability to invade more southerly regions of China has continued since it first invaded northern China thirty years ago. A risk assessment showed that the regions within 21.20° N −46.33° N, 97.80° E −132.11° E contain potentially suitable habitats for *H. cunea* [44]. This moth currently has a known distribution of latitude ranging from approximately 32° N to 40° N in China, but it has not expanded to more southern latitudes. However, our results revealed that most of the individuals from the first generation pupae collected in Qingdao (temperate region) emerged successfully in the climatic conditions of Nanchang (subtropical region). It is worth noting that adult emergence occurred over a very long period from mid-July to late September, showing a dispersed pattern of emergence [45]. Such a pattern may play an important role in determining reproductive success in this moth if it invades southern China. Those individuals emerging in July or early August may not reproduce successfully because their offspring (larvae) will suffer heavy mortality under high temperatures during the summer, as shown in our experimental rearing temperature of 31°C, where 100% of the larvae died before pupation. Those individuals emerging between late August and late September potentially could reproduce because the temperature during autumn in Nanchang is very suitable for larval growth. Therefore, survival of the population in Nanchang may depend on those individuals who have entered dormancy during the summer. If this is the case, there will be two generations if this moth invades Nanchang, that is, one generation in spring and another in autumn. Moreover, our results showed that some diapausing pupae of *H. cunea* successfully overwintered in Nanchang and the adults emerged between late April and mid-May (Fig. 7); whereas the host plants of *H. cunea* in Nanchang generally begin to sprout in mid-March and flush in mid-April. Therefore, adult emergence is fairly well-synchronized with the availability of larval host plants, which could allow this moth to establish breeding populations in subtropical regions of China.
